# Formulation and evaluation of *Ocimum sanctum Linn* containing carboxymethylcellulose and sorbitol based hydrogel

**DOI:** 10.6026/97320630019546

**Published:** 2023-05-31

**Authors:** P Swarna Meenakshi, Ramamurthy Jaiganesh, Rajalakshmanan Eswaramoorthy

**Affiliations:** 1 Department of Periodontics, Saveetha Dental College and Hospitals, Saveetha Institute of Medical and Technical Sciences (SIMATS)Saveetha University, Chennai

**Keywords:** Hydrogel, Ocimum sanctum, sorbitol;, periodontitis

## Abstract

It is of interest to formulate a hydrogel consisting of *Ocimum sanctum Linn*, sorbitol, and carboxymethyl cellulose and to evaluate the physical properties of the hydrogel. The hydrogel was prepared by dissolving *Ocimum sanctum Linn* extract into the mixture containing sorbitol and carboxymethyl cellulose. The formulation was further evaluated for its swelling index, contact angle, in -vitro release properties, and surface analysis using atomic force microscopy. The swelling index showed a significant increase in weight from 1st hr to the 84th hour which is 11.1% and 15.8% respectively. The contact angle test showed a value of 72.81° and 75.99° respectively. In vitro drug release showed a burst release till the 6th day followed by a sustained release till the 20th day. Atomic force microscopy revealed smooth and consistent surface topography with a mean size of 51µm in diameter which depicts that the particles are well dispersed throughout the hydrogel matrix. Data show that hydrogel containing *Ocimum sanctum Linn* extract, sorbitol, and carboxymethyl cellulose could be an efficient economic primeval substitute that is non-toxic, natural, and structured for clinical application.

## Background:

Commonly referred to as hydrogels, hydrophilic gels are networks of polymer chains that occasionally appear as colloidal gels with water as the primary dispersion medium. Researchers have offered numerous definitions of hydrogels throughout the years, the most common of which is a hydrogel, which is a water-swollen, cross-linked polymeric network generated by the simple reaction of one or more monomers. Another description is a polymeric material that can swell and retain a substantial amount of water within its structure but cannot dissolve in water [1]. Because of their remarkable promise in a wide range of applications, hydrogels have attracted substantial investigation over the last 50 years. They are extremely pliable, similar to actual tissue, due to their high water content. Hydrogels absorb water due to hydrophilic functional groups attached to the polymeric backbone, but they are resistant to disintegration due to cross-links between network chains. Hydrogels are two- or multi- component systems composed of a three-dimensional network of polymer chains and water that fills the gap between macromolecules.

Ayurveda, the world's oldest medical system and a science of life, tackles health and disease holistically, focusing on conserving and sustaining good health and preventing disease via healthy lifestyle choices [2]. Ayurveda's use of medicinal and culinary plants draws on India's vast variety in ways that no other medical system does; nevertheless, none of the herbs utilised have the same reputation as tulsi or holy basil (Ocimum sanctum). Tulsi is an aromatic shrub of the Lamiaceae (tribe ocimeae) basil family that originated in north central India but currently thrives locally throughout the eastern global tropics [2][3]. Tulsi is revered in Ayurveda as a "elixir of life" unparalleled in both medical and spiritual properties, and is known as "The Incomparable One," "Mother Medicine of Nature," and "The Queen of Herbs" [4]. Tulsi is a potent adaptogen with a distinct combination of pharmacological properties that promote well-being and resilience [5]. The medicinal benefits of Tulsi have been thoroughly researched in hundreds of scientific investigations, including in vitro, animal, and human trials [5]. According to these studies, Tulsi has a unique combination of antimicrobial (including antibacterial, antiviral, antifungal, antiprotozoal, antimalarial, and anthelmintic properties), antidiarrheal, antioxidant, anti-cataract, anti-inflammatory, chemo-preventive, radio-protective, hepato-protective, neuro-protective, cardio protective, and anti-diabetic properties [6][7] [8][9].

Sorbitol, also known as glucitol, is a sugar alcohol with a pleasant flavour that the human body digests slowly. It can be made by reducing glucose, which transforms the transformed aldehyde group (CHO) into a primary alcohol group (CH2OH). The majority of sorbitol is produced synthetically from potato starch, but it can also be found naturally in apples, pears, peaches, and prunes. Sorbitol is frequently used in the cosmetics sector and is thought to have a low toxicity and environmental impact [10]. A recent study used a dibenzylidene sorbitol framework to construct pseudoephedrine, preventing the active pharmaceutical ingredients from being easily extracted for usage in illicit substances [11]. Sorbitol gels were pH-tolerant and effective at absorbing large volumes of polluted particles [12]. Cellulose is the most plentiful and least expensive natural polymer on the planet, with numerous important features such as biocompatibility, high moduli, low density, hydrophilicity, and high modification potential [12]. As a result, the concept of mixing sorbitol, cellulose, and occimum sanctum piqued our interest.

Periodontitis is an inflammatory disease of the teeth's supporting tissues caused by one or more microorganisms, resulting in the gradual loss of periodontal ligament and alveolar bone, as well as periodontal pocket development, gingival recession, or both [12]. These modifications are linked to pathologic tooth migration [13] [14]. Perioceutics, which encompasses the administration of therapeutic drugs by systemic and local routes as an adjuvant to mechanical therapy, has transformed the field of periodontal therapy [15] [16]. Nonsurgical periodontal therapy is always the key to success of periodontal treatment. As a result, in addition to scaling and root planing, anti-microbial medicines play an important role in successful periodontal care [17]. Local drug delivery agents currently on the market include irrigation systems, fibers, gels, and strips. Hydrogels have been widely used as scaffolds in regenerative medicine and as a sustained release in periodontitis treatment thereby preventing the growth of microorganisms [17-18]. Therefore, it is of interest to formulate a hydrogel using *Ocimum sanctum Linn*, sorbitol, and carboxymethyl cellulose and assess the physicochemical properties of the hydrogel to use it as a local drug delivery in periodontitis patients.

##  Material and Methods:

## Preparation of gel:

## Preparation of supercritical fluid:

**Figure 1:**
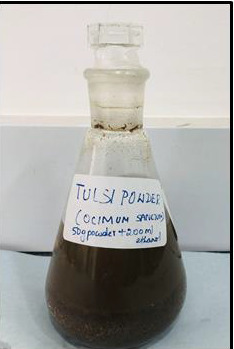
Tulsi powder was mixed with ethanol

For 48 hours, 50gms of Tulsi Powder was steeped in 1000mL of Ethyl alcohol (Figure 1). Whartman's filter is used to filter this mixture. The maceration process was used to create this ethanolic extract of *Ocimum sanctum Linn*. The filtered liquid was evaporated in a water bath at 65°C for 30 minutes to produce supercritical fluid, which was then kept in a suitable container at the right temperature.

## Preparation of *Ocimum sanctum Linn* gel:

**Figure 2:**
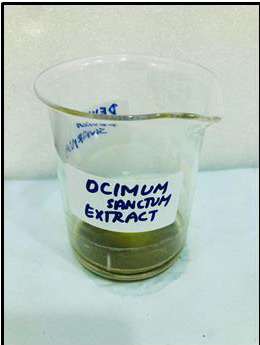
*Ocimum sanctum*extract

Carbopol was soaked in purified water containing 0.2% w/v sodium benzoate overnight. Using a tissue homogenizer carboxymethylcellulose (CMC) solution was mixed with propylene glycol. 2ml of tulsi extract (supercritical fluid extract) was homogenised in CMC solution. This drug solution was then homogenised and added to carbopol solution. To neutralise the pH, triethanolamine was added. Carboxymethyl cellulose gel was made by dissolving 2 gms of carboxymethyl cellulose in 100 ml of distilled water and refrigerating it for 24 hours. Sorbitol (0.5 gm) was combined with occimum sanctum gel (0.5 gm) (Figure 2). This combination was mixed with 100ml of carboxymethyl cellulose and refrigerated for 24 hours. 30 cc of this mixture was placed onto petri dishes and left at 37°C overnight.

## Swelling Index:

Swelling test was done to measure the amount of water content of the *Ocimum sanctum Linn* hydrogel, wherein 10mg of freeze- dried hydrogels were placed in 100µl of phosphate buffered solution at 37°C. These hydrogels were taken from the PBS solution after 24 hours, dabbed with kimwipe wipers to remove any extra water on the surface, weighed, and reintroduced into the buffer solution. This method was repeated for a total of 84 hours.

Swelling Ratio (SR) = ((w2-w1)/(w1) x100 %

W2 and W1 are the weight before swelling and after swelling respectively.

In vitro drug release: The drug release of *Ocimum sanctum Linn* gel in vitro was measured as follows. In 1 ml of phosphate buffer solution, 5mg of hydrogel was dissolved. To imitate the physiological setting, the hydrogel dispersion was incubated at 37°C with mechanical agitation. 1ml of release solution was collected by centrifuge tubes at regular intervals and replaced with an equal volume of fresh phosphate buffer solution. Each time, triplicate wells were used. The absorbance measurements were taken at 450nm and recorded by a microtiter plate reader under a UV spectrophotometer. The log-logit approach was used with the GraphPad prism program and a standard curve to compute the gel concentrations measured at different intervals.

## Contact angle test:

A goniometric approach was used to measure advancing and receding contact angles. The contact angle was measured using a K100 Force Tensiometer. Advancing (right and forward) measurements were taken by making a 0.46gm drop of water at the end of a needle, contacting it to a test surface, and then adding water to the drop in 0.25mg increments until the drop edge was observed to advance by an operator using a digital imaging system. Receding angle measurements were taken (left and backward) by draining water through the needle until the drop edge receded.

## Atomic Force Microscopy:

The surface characteristics, including height and distance measurements, roughness calculation, grain, and particle analysis, and 3D visualization can be done using AFM. AFM was done using NANOSURF C3000 which measures the distance between the particles and the images are stitched together into a single image by Nanosurf report expert post processing software.

## Results:

## Swelling index:

**Figure 3:**
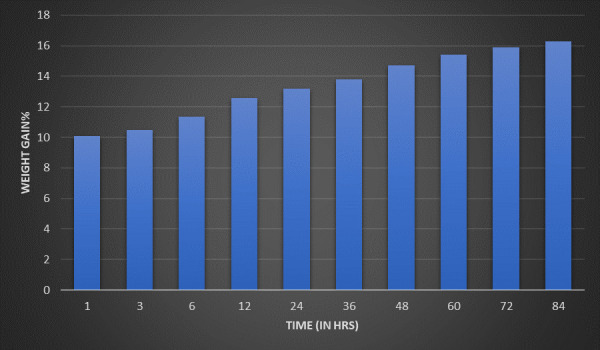
Swelling index - shows the percentage of swelling (weight gain) with respect to time in hours

The hydrogel's swelling index was measured after 1 hour and up to 84 hours. Our preparation demonstrated accelerated swelling due to the porous nature of the hydrogels providing a large surface area and permitting rapid uptake of the solvent, but considerable swelling was not observed after 6 hours and almost remained steady after 6 hours. Swelling index after 1 hr, 6hr, 84hrs were 11.1%, 13.7%, and 15.8% respectively (Figure 3). This finding could be accredited to the presence of sorbitol and carboxymethyl cellulose as both components are viscous in their behavior. We can infer from the above data that the swelling ratio increases, but not much, indicating that there was not much degradation of the hydrogel, boosting nutrition to the tissues and decreasing inflammation.

## In vitro drug release:

**Figure 4:**
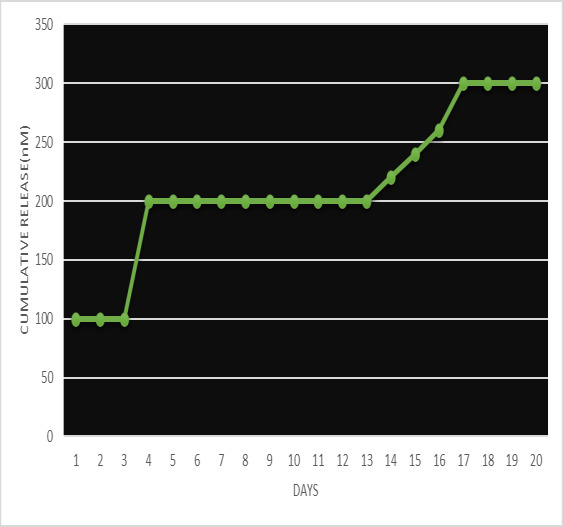
Shows cumulative release profiles of *Ocimum sanctum Linn* hydrogel microspheres in PBS at 37°C for 20 days. Burst release was seen between 1- 3rd day. After a 3 day burst release, the average daily release of hydrogel was 200-210nm till the 13th day followed by a burst release of 250-280nm till the 17 th day and a sustained release of 300 nm from 18- 20 days.

The release properties of the hydrogel were measured by a UV spectrophotometer. After a 3 day burst release (90-100nM), the average daily release of the hydrogel was in the range of 200-210 nM depicting a sustained release till the 13th day followed by a burst release(250-280nM) till the 17th day and a sustained release of 300nM from 18-20 days (Figure 4). The results revealed a concentration-dependent increase in the amount of sorbitol, *Ocimum sanctum Linn* and carboxymethyl cellulose.

## Contact angle test:

**Figure 5:**
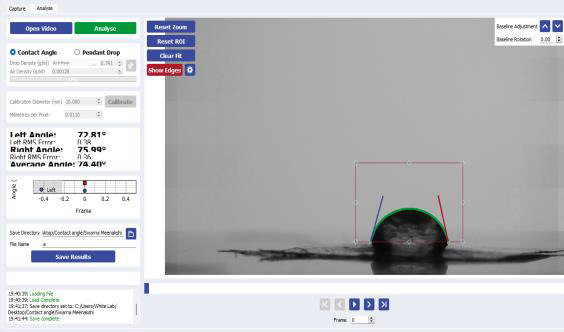
Left contact angle shows 72.81° and right contact angle shows 75.99° which reveals the hydrogel is hydrophilic in nature

The contact angles on the left and right sides were 72.81° and 75.99°, respectively (Figure 5). Because the contact angle is smaller than 90°, the *Ocimum sanctum Linn* hydrogel is hydrophilic, which means it can absorb more water and quickly wets the tissues. This demonstrates that the formulated hydrogel will rapidly moisten the tissues, implying that the hydrogel will adhere to the tissues effectively.

## Atomic force microscopy:

**Figure 6:**
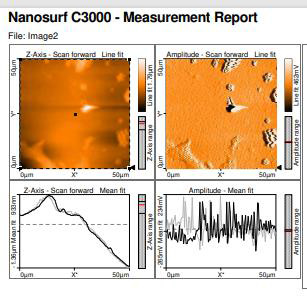
Reveals a surface roughness of 119.32nm and the mean size of 80% of the particles is in the range of 50-71µm; the mean size was 51µm in diameter which depicts that the particles are well dispersed throughout the hydrogel matrix and morphological changes of the hydrogel were consistent throughout this period of 20 days. No breakage of particles was observed during the 20 days of release.

This reveals a surface roughness of 119.32nm and the mean size of 80% of the particles is in the range of 50-71µm (Figure 6). The mean size of the particle was 51µm in diameter which depicts that the particles are well dispersed throughout the hydrogel matrix and morphological changes of the hydrogel were consistent throughout this period of 20 days. No breakage of particles was observed during the 20 days of release. The AFM shows that the combination of sorbitol, *Ocimum sanctum Linn* extract, and carboxymethyl cellulose are bonded well to each other indicating that it will not degrade more readily. dispersed throughout the hydrogel matrix and morphological changes of the hydrogel were consistent throughout this period of 20 days. No breakage of particles was observed during the 20 days of release.

## Discussion:

We created a sustained release mechanism for the *Ocimum sanctum Linn* hydrogel combined with sorbitol and CMC in this study. Atomic force microscopy revealed that the hydrogel's surface topography was smooth and consistent throughout the degrading process, with a minimum surface roughness of 119.32nm. Previous studies have found that the optimal size of microspheres for intra-articular injection in rats is 35-105 µm, with no adverse effects [19]. The surface analysis results revealed that more than 80% of the composed microspheres were 50-71µm in diameter, specifying that the hydrogel particles may not be toxic and harmful to the gingival tissues and may be a pertinent tool for administration into the gingival tissues. The present composition uses tulsi and sorbitol, CMC owing to execute their best antimicrobial and regeneration properties as evident from previous literature [20].

Hydrogels have been exclusively used because of their sustained delivery of a variety of local therapeutic agents [21, 17]. Sorbitol, a natural polymer, and sweetener have been used in our study, proves to its sustained release in therapeutics and garb of biological mechanisms and mechanical strengths. Swelling index was measured for the preparation from 1st hr till 84 hrs of gel formulation. It was observed that our preparation manifested expeditious swelling due to the penetrable nature of the hydrogels providing enormous surface area permitting rapid absorption of the solvent but it was noticed that the swelling did not increase significantly divulging the fact that the hydrogel will not degrade readily and will help in enhancing the nutrition to the tissues and helps in cell migration thereby reducing the inflammation.

Data shows an appreciable contact angle proving the hydrogel formulation to be hydrophilic in nature thus enhancing the adhesion of the hydrogel to the tissues. Our preparation showed supercilious and substantial sustained drug release, which could be imputed to the property of natural polymer and sweetener sorbitol used in this study. This infers that the hydrogel is less toxic because sorbitol does not cause an acidic environment in the oral cavity and is naturally occurring in fresh fruits like mangoes, apples, and peaches which make the formulation superior in quality. This sorbitol also attributes to the viscosity of the formulated hydrogel. In another study where sorbitol-based hydrogel was formulated for therapeutic drug monitoring, it has been proved that the presence of these sugar alcohols within a hydrogel system functions as a hyper-osmolyte that endowed the hydrogel systems with additional osmotic pressure to enable rapid and extensive swelling in the presence of physiological fluid [22] [23]. In addition, the presence of additional -OH (hydroxyl) groups from the sugar alcohols enabled even more hydrogen bonding to be formed between the hydrogel and water, thus expanding the extraction capability and water holding capacity of the overall system [24]. Furthermore, CMC, which acts as a binder on the polymeric surface, has been used in this study which increases the wettability of viscosity of the formulated hydrogel [25].

In another study, it has been shown that CMC-based hydrogels such as the CMC-PVA hydrogel are used in facial masks for improving moisture or water retention capacity [26][27]. This makes our study reliable because as the water retention capacity increases the degradation decreases and enhances the nutrition of the tissues. Non-surgical periodontal therapy plays a vital role in the success of periodontal treatment. Therefore as a measure to enhance and make the non-periodontal therapy efficient, apart from scaling and root planing other anti-microbial agents like irrigation systems, hydrogels, and strips are being used [28]. Herbal interventions have also gained attention and have been incorporated into the treatment of periodontal therapy [29]. Thus, the hydrogel which has been formulated in our study consisting of *Ocimum sanctum Linn*, sorbitol, and carboxymethyl cellulose can be used as a local drug delivery agent in periodontitis patients before proceeding with surgical periodontal therapy.

## Conclusion:

We were able to, fortunately, formulate this hydrogel containing *Ocimum sanctum Linn* extract, sorbitol, and carboxymethyl cellulose and assessed the physical properties of the same. The findings of our study could be an efficient economic primeval substitute that is non-toxic, natural, and structured for clinical application. Further studies are needed before using this novel formulation as a local drug delivery agent in patients with periodontal diseases.
